# Supplementary material for: A Prognostic Model Based on Nutritional Risk Index in Operative Breast Cancer
Source: Nutrients. 2022 Sep 14;14(18):3783. doi: 10.3390/nu14183783 (PMC9502262; doi:10.3390/nu14183783)
Supplement: Supplementary file 1 [file nutrients-14-03783-s001.zip › nutrients-1888492-supplementary.pdf]

Supplementary Figure Legends

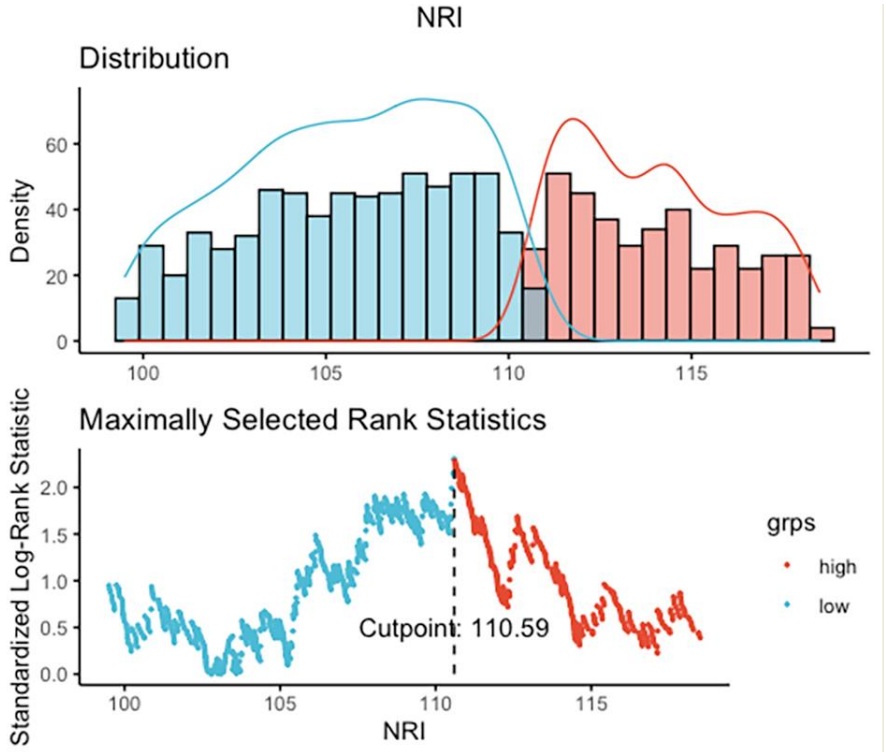

Figure S1. The optimal cut-off of NRI for overall survival in the whole cohort by using maximally selected rank statistics.

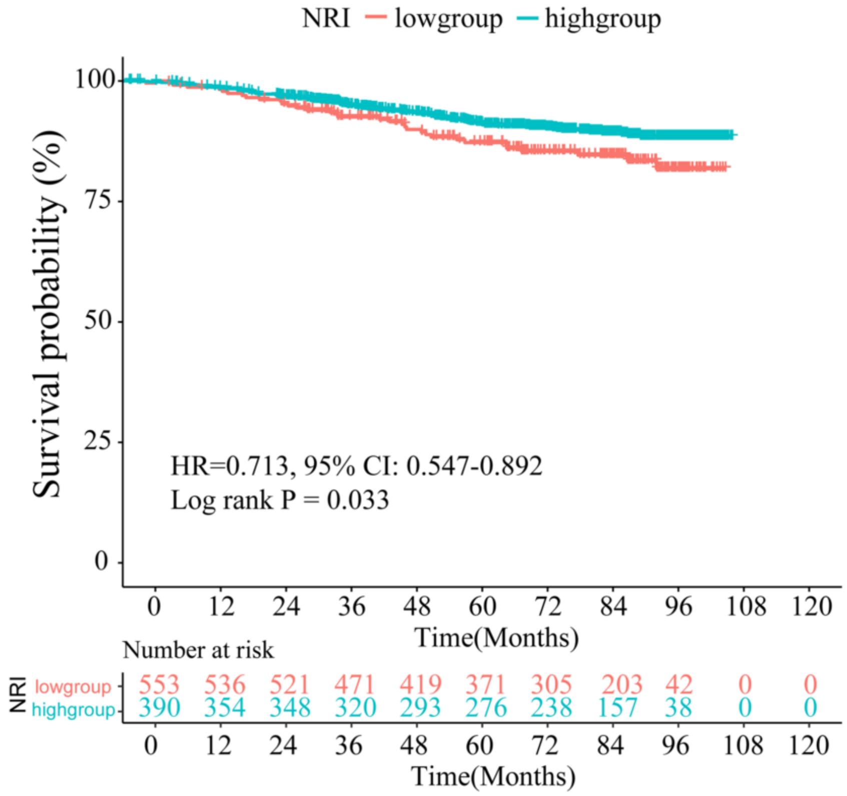

Figure S2. Kaplan-Meier survival curves of breast cancer patients after surgery between the high-NRI group and low-NRI group in the training set.

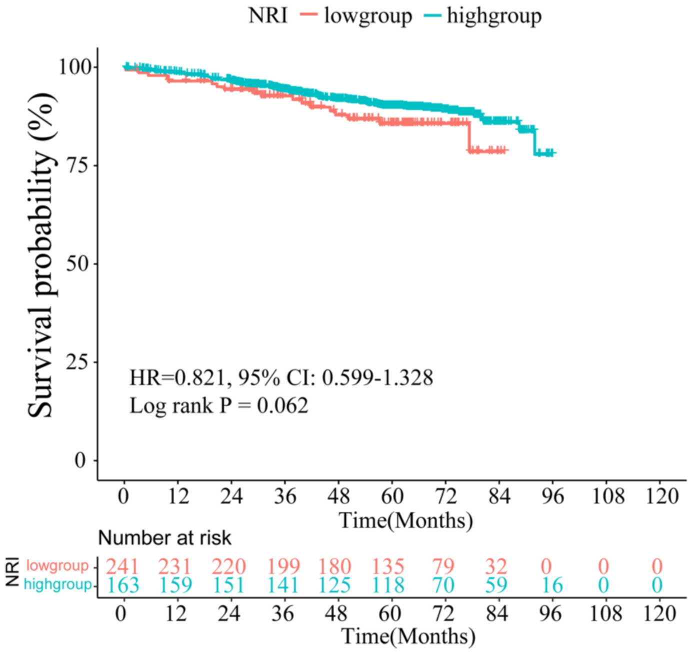

Figure S3. Kaplan-Meier survival curves of breast cancer patients after surgery between the high-NRI group and low-NRI group in the validation set.

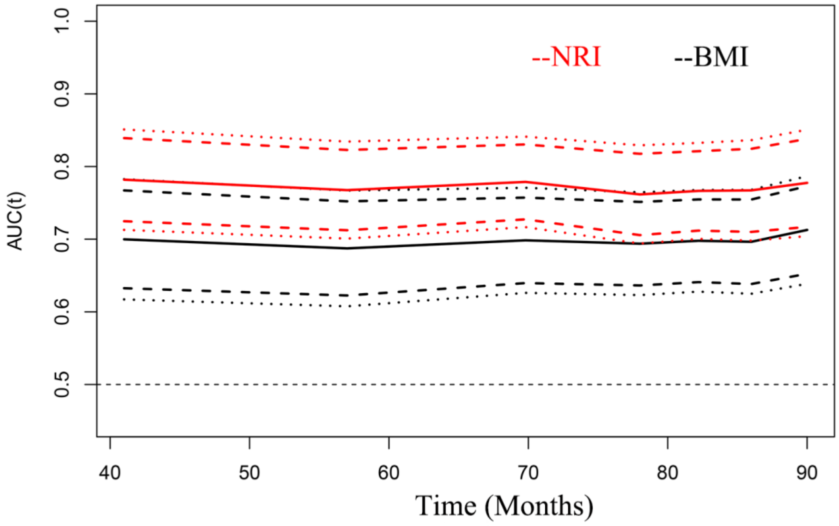

Figure S4. Time-dependent ROC curve compared with NRI and BMI.

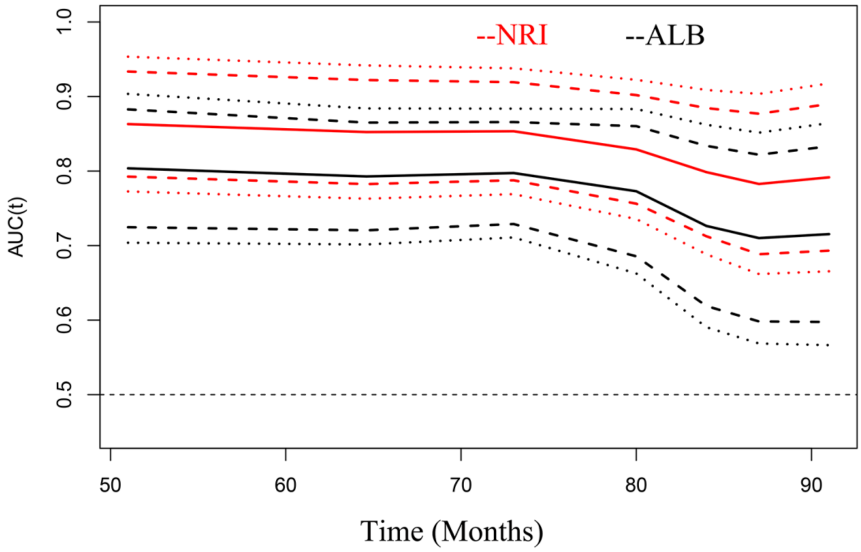

Figure S5. Time-dependent ROC curve compared with NRI and ALB.

Table S1. Univariate analysis and multivariate regression analysis of overall survival by Cox regression analysis in the training set.

Characteristics	Univariate analysis Hazard Ratio (95% CI)	*P*	Multivariate analysis Hazard Ratio (95% CI)	*P*
Age (years)				
≤50	1		-	-
>50	1.556 (0.742-1.814)	0.513	-	-
Histopathological Type				
Invasive ductal carcinoma	1		1	
Others	0.408 (0.173-0.735)	0.008*	0.425 (0.203-0.826)	0.005*
Tumor size				
≤2cm	1		1	
>2cm	3.345 (1.613-4.198)	<0.001*	2.420 (1.251-2.833)	0.032*
Lymph node status				
No lymph node metastasis	1		1	
With lymph node metastasis	4.890 (4.111-7.535)	<0.001*	4.923 (3.775-5.681)	<0.001*
ER status				
Negative	1		-	-
Positive	0.549 (0.372-1.648)	0.580	-	-
PR status				
Negative	1		1	
Positive	0.516 (0.327-0.814)	<0.001*	0.501 (0.402-0.699)	0.008*
HER-2 status				
Negative	1		1	

Positive	1.835 (1.146-2.879)	0.003*	1.546 (0.772-1.832)	0.505
Ki-67				
≤14%	1		1	
>14%	2.439 (1.576-3.480)	<0.001*	1.739 (1.330-2.501)	0.011*
Adjuvant chemotherapy				
No	1		1	
Yes	1.658 (1.246-2.834)	0.035*	2.138(0.619-3.114)	0.087
Radiotherapy				
No	1		1	
Yes	1.836 (1.411-2.815)	<0.001*	1.961 (0.676-2.230)	0.157
Endocrine therapy				
No	1		-	-
Yes	0.653 (0.475-1.162)	0.124	-	-
Target therapy				
No	1		-	-
Yes	1.158 (0.506-2.201)	0.973	-	-
NRI				
≤110.59	1		1	
>110.59	0.713 (0.547-0.892)	0.033*	0.736(0.512-0.923)	0.041*

Abbreviations: NRI: Nutritional Risk Index; ER: Estrogen Receptor; PR: Progesterone Receptor; HER2: Human Epidermal Growth Factor Receptor-2. Patients were staged according to the 8th American Joint Committee on Cancer-Tumor, Node, and Metastases (AJCC-TNM) stage. The symbol "*" was indicate that the P value is less than 0.05.

Table S2. Univariate analysis and multivariate regression analysis of overall survival by Cox regression analysis in the validation set.

Characteristics	Univariate analysis Hazard Ratio (95% CI)		Multivariate analysis Hazard Ratio (95% CI)	
		P		*P*
Age (years)				
≤50	1		-	-
>50	1.498 (0.813-1.776)	0.549	-	-
Histopathological Type				
Invasive ductal carcinoma	1		1	
Others	0.426 (0.205-0.712)	0.009*	0.439 (0.252-0.736)	0.006*
Tumor size				
≤2cm	1		1	
>2cm	3.571 (1.730-4.188)	<0.014*	2.698 (1.483-3.106)	0.040*
Lymph node status				
No lymph node metastasis	1		1	
With lymph node metastasis	4.912 (5.563-6.365)	<0.001*	5.357 (3.406-6.224)	<0.001*
ER status				
Negative	1		-	-
Positive	0.549 (0.372-1.648)	0.580	-	-
PR status				
Negative	1		1	
Positive	0.582 (0.367-0.936)	<0.002*	0.698 (0.231-0.803)	0.015*
HER-2 status				
Negative	1		1	

Positive	1.652 (1.188-2.379)	0.016*	1.890 (0.835-2.307)	0.433
Ki-67				
≤14%	1		1	
>14%	3.701 (1.871-4.205)	<0.002*	3.379 (1.901-3.961)	0.006*
Adjuvant chemotherapy				
No	1		-	-
Yes	1.818 (0.794-2.650)	0.063	-	-
Radiotherapy				
No	1		1	
Yes	1.532 (1.129-3.190)	<0.001*	2.506 (0.915-2.921)	0.248
Endocrine therapy				
No	1		-	-
Yes	0.502 (0.429-1.237)	0.205	-	-
Target therapy				
No	1		-	-
Yes	1.362 (0.528-2.447)	0.791	-	-
NRI				
≤110.59	1		-	-
>110.59	0.821 (0.599-1.328)	0.062	-	-

Abbreviations: NRI: Nutritional Risk Index; ER: Estrogen Receptor; PR: Progesterone Receptor; HER2: Human Epidermal Growth Factor Receptor-2. Patients were staged according to the 8th American Joint Committee on Cancer-Tumor, Node, and Metastases (AJCC-TNM) stage. The symbol "*" was indicate that the P value is less than 0.05.
